# Identifying the Age Cohort Responsible for Transmission in a Natural Outbreak of *Bordetella bronchiseptica*

**DOI:** 10.1371/journal.ppat.1001224

**Published:** 2010-12-16

**Authors:** Gráinne H. Long, Divya Sinha, Andrew F. Read, Stacy Pritt, Barry Kline, Eric T. Harvill, Peter J. Hudson, Ottar N. Bjørnstad

**Affiliations:** 1 Center for Infectious Disease Dynamics, Department of Biology, The Pennsylvania State University, University Park, Pennsylvania, United States of America; 2 Veterinary and Biomedical Sciences, Department of Biology, The Pennsylvania State University, University Park, Pennsylvania, United States of America; 3 Fogarty International Center, National Institutes of Health, Bethesda, Maryland, United States of America; 4 Covance Research Products Incorporated, Denver, Pennsylvania, United States of America; Emory University, United States of America

## Abstract

Identifying the major routes of disease transmission and reservoirs of infection are needed to increase our understanding of disease dynamics and improve disease control. Despite this, transmission events are rarely observed directly. Here we had the unique opportunity to study natural transmission of *Bordetella bronchiseptica* – a directly transmitted respiratory pathogen with a wide mammalian host range, including sporadic infection of humans – within a commercial rabbitry to evaluate the relative effects of sex and age on the transmission dynamics therein. We did this by developing an *a priori* set of hypotheses outlining how natural *B. bronchiseptica* infections may be transmitted between rabbits. We discriminated between these hypotheses by using force-of-infection estimates coupled with random effects binomial regression analysis of *B. bronchiseptica* age-prevalence data from within our rabbit population. Force-of-infection analysis allowed us to quantify the apparent prevalence of *B. bronchiseptica* while correcting for age structure. To determine whether transmission is largely within social groups (in this case litter), or from an external group, we used random-effect binomial regression to evaluate the importance of social mixing in disease spread. Between these two approaches our results support young weanlings – as opposed to, for example, breeder or maternal cohorts – as the age cohort primarily responsible for *B. bronchiseptica* transmission. Thus age-prevalence data, which is relatively easy to gather in clinical or agricultural settings, can be used to evaluate contact patterns and infer the likely age-cohort responsible for transmission of directly transmitted infections. These insights shed light on the dynamics of disease spread and allow an assessment to be made of the best methods for effective long-term disease control.

## Author Summary

A lack of understanding regarding determinants of infectious disease transmission has hindered improved disease control efforts. Here we had the unique opportunity to study the natural transmission of the respiratory pathogen *Bordetella bronchiseptica* within a commercial rabbitry. *B. bronchiseptica* is a directly transmitted gram-negative bacterium belonging to the genus *Bordetella*, which also comprises *B. pertussis* and *B. parapertussis*, the etiological agents of whooping cough in humans. In this study we estimated the importance of rabbit sex, age and social group on disease spread. To do this we first outlined a set of hypotheses about how natural *B. bronchiseptica* infections may be transmitted between rabbits. We then discriminated between these hypotheses by estimating the rate at which susceptible individuals acquire infection (or force-of-infection) using *B. bronchiseptica* age-prevalence data. The importance of social structure in disease spread was then evaluated using random-effect binomial regression. Our results support young weanlings as the age cohort primarily responsible for *B. bronchiseptica* transmission and demonstrate that easy to collect age-prevalence data can be used to infer the likely age-cohort responsible for disease transmission. Such insights shed light on the dynamics of disease spread and allow an assessment to be made of the best methods for effective disease control.

## Introduction

Containing and ultimately eliminating infectious disease remains a central goal for many animal and public health officials. Dissecting disease transmission – in terms of identifying the routes and potentially heterogeneous rates of disease spread [1] – is an essential step in devising or optimizing intervention strategies aimed at pathogen eradication [1], [2]. This is because heterogeneities in transmission that arise due to for example age- or sex-specific differences among individuals [2], [3] can greatly affect invasion and eradication criteria [1]. Unfortunately, precise measurements of transmission remain elusive due to the immense difficulties associated with identifying the nature of a potential contact, the probability of infection given a contact [4] and important drivers of heterogeneities in transmission [2], [3], [5]. A key reason for these difficulties is that transmission events are rarely observed directly, with some notable exceptions [6].

One useful approach that can shed partial light on the transmission process is to measure the force-of-infection (FOI: λ), or the per capita conversion rate of susceptible hosts [7]. The simplest way to think about the FOI, is that over a short interval of time – say from time t to t+Δ – the probability that a disease negative individual becomes disease positive is λΔ. The most popular way to estimate λ is through use of the observed age-specific prevalence (or the proportion of individuals that are disease positive in a cross-sectional sample), due to the ease with which it is measured in most populations [8]. Indeed, FOI estimates have been calculated from age-prevalence data for human, and to a lesser extent, wildlife infections [3], [9], [10], [11], [12], [13], [14], [15], [16], [17], [18], [19]. Estimating the FOI can help identify those age-classes responsible for transmission and evaluate the relative effects of each group on overall transmission. Here we evaluate the relative effects of sex, age and social structure on the transmission dynamics of the respiratory pathogen *Bordetella bronchiseptica* within a commercial rabbitry of New Zealand White (NZW) rabbits. In doing so we illustrate how analysis of age-prevalence data can be used to estimate the age-specific FOI. The importance of social organization in *B. bronchiseptica* transmission is also considered. To test for litter-based transmission events – for example, from mother to offspring or between siblings – we checked for significant correlation among the fate of siblings by using a litter-based random-effects binomial regression to estimate the importance of horizontal *versus* pseudovertical transmission [20], [21]. The statistical tools we employ here are general and can be applied to a range of directly transmitted medical and veterinary diseases to help shed light on the dynamics of disease spread and allow an assessment to be made of the best methods for effective long-term disease control.

**Figure 1:**
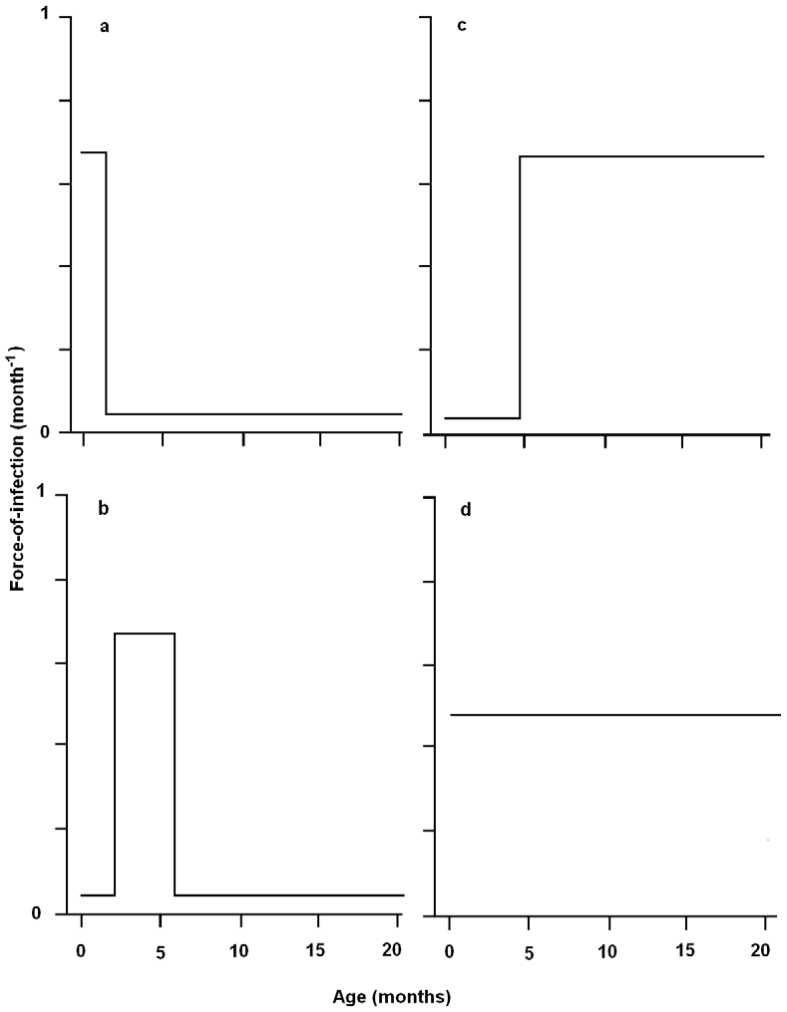
Schematic representing the possible age-specific force-of-infection for transmission of *Bordetella bronchiseptica* infection to New Zealand White rabbits in a commercial rabbitry. Possible routes of transmission include (*a*) the pseudo-vertical route from mother to new born offspring until the age of weaning (∼4 to 5 weeks), (*b*) horizontally between siblings when re-housed at age of independence (from 4 to 5 weeks until 5 to 6 months of age), (*c*) horizontally at time of breeding (from 5 to 6 months onwards) and (*d*) transmission from birth due to environmental contamination.

The *Bordetella* genus contains three closely related gram-negative bacteria that cause respiratory infections in humans and other mammals [22]. Whereas *B. pertussis* and *B. parapertussis* largely infect humans and cause the acute respiratory disease known as whooping cough [23], *B. bronchiseptica* typically causes chronic infections in a wide range of mammals [24]. Indeed *B. bronchiseptica* infection is often endemic in agricultural settings – including commercial rabbitries [25], [26] – where rapid spread and persistent infection make it difficult to control [23]. Despite its widespread nature, there is a paucity of data describing the epidemiology of *B. bronchiseptica* in terms of both the main route(s) of, and likely cohort(s) responsible for disease transmission. As a respiratory infection, the major physical route of transmission is oral-nasal via direct aerosol droplets [27], [28]. Based on the published literature [26], [27], [29], we propose a set of plausible routes of transmission within a commercial rabbitry would include:

- the pseudo-vertical oral-nasal route from mother to new born offspring before the age of weaning (4 to 5 weeks of age), with transmission occurring during suckling or grooming (figure 1*a*);
- horizontal between sibling transmission when re-housed at age of independence (4 to 5 weeks until 5 to 6 months of age) via routine social behavior such as den-sharing or grooming (figure 1*b*);
- horizontally at time of breeding (5 to 6 months onward: figure 1*c*)
- constant from birth via environmental contamination (figure 1*d*).

These (not mutually exclusive) possible routes of transmission may result in the prevalence of infection changing with age in different ways which can be related to different underlying hazard models/FOI patterns (Figure 1). As we will explore below, each of these transmission possibilities translates into a specific prediction which can be tested using our statistical framework. To identify the parsimonious hypothesis, we applied a piece-wise constant model for the age-specific FOI [9], [11], [15]. Since *B. bronchiseptica* is an endemic persistent infection [23], we used a catalytic framework (which assumes a one-way flow from susceptible to infected). The importance of sex and location (facility building) and time of sampling on FOI estimates was also determined. We considered the importance of social mixing and organization in *B. bronchiseptica* transmission using random effect logistic regression estimates to control for litter as confounding variable in transmission models. In parallel, we took a molecular epidemiological approach to investigate whether strain-specific differences existed in the epidemiological pattern of infection [30], [31].

## Methods

### Ethics statement

All protocols involving rabbits were approved by the Institutional Animal Care and Use Committee (IACUC) at the Pennsylvania State University according to the guidelines of the American Association for Laboratory Animal Science.

### Rabbit hosts and facility

This study was conducted at a commercial rabbitry which breeds NZW rabbits. The rabbitry comprised of three separate animal breeding buildings (Table 1; buildings A - C). Background health checks – in the form of comprehensive monthly pathology reports testing for >17 pathogens – have been carried out since January 2003 (n = 2 to 4 rabbits/month/building). These reports show that *B. bronchiseptica* has been endemic in the rabbitry since testing began and that of the other pathogens screened, only non-pathogenic *Eimeria* species (intestinal coccidia) are occasionally isolated. Importantly, our rabbitry is *Pasteurella multocida* free – infection with this respiratory pathogen has long been associated with upper respiratory disease (URD) in rabbits [32] – with no URD reported in the last 30 years.

**Table 1:** Rabbitry Buildings.

Description	Building A	Building B	Building C
Total population size (n)	∼14-15,000	∼3-4,000	∼6-7,000
Shower in barrier	yes	yes	yes
Caging system	3-tiered stationary	Rolling racks	3-tiered stationary
Ventilation	Tunnel^2^	Tunnel^2^	HVAC^1^
Cage cleaning	Pressure washer	Rack washer (180°F)	Pressure washer

Description of each of the three buildings which comprise the rabbitry. All rabbits were New Zealand Whites. Colonies have been closed for the past ∼30 years, with 8 family breeding lines to maintain rabbit genetic diversity.

1 HVAC (heating, ventilation and air conditioning): Regulates humidity and temperature.

2 Tunnel: use exhaust fans to pump air from end to end like a wind tunnel.

Kits were weaned at 4–5 weeks of age and ‘weanlings’ segregated by sex and co-housed in sibling pairs. Rabbits of good breeding stock were selected as ‘future breeders’ and housed in pairs. The remaining ‘stock’ rabbits were housed singly and sold at 8–10 weeks old. ‘Breeder’ rabbits were initially bred at 5 or 6 months of age for females and males respectively. Breeders were, housed individually and rebred when litters were weaned. For all rabbits included in this study, the date of sampling, building and rabbit identification number were ascertained along with nasal swab (BD sterile swab, product # 220518).

### Sampling strategies

A total of eight sampling efforts were carried out from November 2006 to September 2008, culminating in the collective nasal swabbing of 602 rabbits total. All nasal swabs were streaked onto Bordet-Gengou (BG) agar (Difco) containing 10% sheep's blood (Hema Resources) with 20 µg/mL streptomycin (Sigma) as soon as possible after collection and incubated at 35°C for ∼3–5 days. The sampling strategies were as follows:

#### Sampling strategy one: force-of-infection

To estimate the FOI, date-of-birth and sex information was obtained for each rabbit along with a nasal swab for culture from 4 cross-sectional sampling efforts across two buildings (Table 1, buildings A & B) that comprised 214 rabbits; 73 does, 44 bucks, 32 male kits, 30 female kits, 16 female future breeders and 19 male future breeders. Litter information was only available for kits. Exact date-of-birth and kit sex information was not collected during sampling strategies two and three (see below). This data could not be obtained retrospectively from the rabbitry and hence prevented the inclusion of these rabbits into FOI analyses.

#### Sampling strategy two: sibling-to-sibling transmission

Nasal swabs of kits (and their does for inclusion in the maternal transmission analyses below) were taken before weaning (∼2- and 4-weeks of age) to ascertain infection status and comprised 160 kits total. At weaning, kits were co-housed in sibling pairs based on their *B. bronchiseptica* infection status as follows; 23 pairs with one sibling positive- and one negative- for *B. bronchiseptica* and 54 pairs where both siblings were negative for *B. bronchiseptica*. As a control, six *B. bronchiseptica* negative siblings were housed solitarily. 4-weeks later, nasal swabs were collected to calculate the number of disease conversion events: specifically, cages containing rabbits converting from *B. bronchiseptica* negative to positive were scored ‘1’, with no change in infection status scored ‘0’.

#### Sampling strategy three: maternal transmission

Nasal swabs of does and their kits taken at weaning (∼4 weeks old) across 3 rabbitry buildings (A, B & C) were used in these analyses and included data from 3 new sampling efforts (n = 208), as well as relevant doe-kit data from the FOI analysis (n = 106 rabbits) and sibling-sibling analysis (n = 180) described above. Thus a total of 86 does and 408 kits were included in the maternal transmission analyses.

### *Bordetella* diversity

We used Multi-Locus Sequence Typing (MLST) analysis [33], [34] to determine the phylogenetic relationships among 90 *B. bronchiseptica* isolates from 4 sampling efforts across three rabbitry buildings, as previously described for *Bordetella.* Briefly, genomic DNA from each isolate was obtained using a DNAeasy Tissue Kit (Qiagen) and nucleotide sequences were determined for internal regions of seven housekeeping genes for all 90 isolates (see the *Bordetella* MLST database at http://pubmlst.org/bordetella). All alleles were double stranded sequenced at The Pennsylvania State University Genomic Sequencing Center and an allele number was assigned to each unique allele sequence. The combination of the allele numbers at the seven loci defines the sequence types (ST) or allelic profile of each strain [33], [35].

### Statistical analysis

#### (i) Force-of-Infection models

All analyses were performed in R version 2.7.0 (http://www.R-project.org) and the fully annotated R code is available as Supporting Information (Text S1). There are several methods for establishing disease foothold in a population; one measure is the FOI (λ), or the per capita infection rate of susceptible hosts. For persistent infections the most popular way to estimate λ is through use of the observed age-specific prevalence, largely due to the ease with which it is measured in most populations. It should be noted that for non-persistent fully-immunizing infections, the FOI can be inferred from age-seroprevalence profiles [8]. Calculating the FOI from age-profiled data is generally referred to as the ‘catalytic framework’ in mathematical epidemiology [7]. In our study, age-specific prevalence data was collected from rabbit nasal swabs and is interval-censored infection-time data such that each individual is either infected (*Y* = 1) or not (*Y* = 0) within a set interval of time.

To estimate the FOI, we make a number of necessary and common assumptions about our data: that no portion of the rabbit population is free from disease exposure; that a perfect test is used (i.e. in our case, that nasal swabbing always detects infection when present); that disease-induced mortality is negligible; and that infection is lifelong (or in the case of age-seroprevalence data, that immunity is lifelong). As *B. bronchiseptica* infections in our rabbit population are chronic and non-virulent, we assume that the infection is irreversible – meaning that infection is lifelong – and that the infection-induced mortality is negligible and can be ignored. For data sets which may violate any of the above assumptions, alternative methods [8], as well as extensions to existing models to incorporate infection processes such as death and periods of passive maternal protection are now available for estimating the age-specific FOI, λ(a) [15]. For a non-immunizing persistent infection such as *B. bronchiseptica,* the age-specific prevalence, *P*(a), is defined by the differential equation 
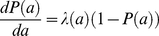
(1)where (1-*P*(a)) is the age-specific proportion of susceptible hosts. In epidemiology, age is considered one of the main risk factors for infection and disease prevalence often shows strong and distinct relationships with age [15]. To illustrate this, suppose individuals are born susceptible. Total time of disease exposure therefore increases with age and thus, disease prevalence appears to be directly linked to population age-structure. Our population is an animal breeding facility and so is skewed towards younger age-classes, which suffer less disease-exposure time and therefore possibly lower overall disease prevalence. Equation (1) leads to the following prediction for how disease prevalence should depend on age: 
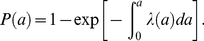
(2)


Equation (2) presents a catalytic model [7] that incorporates how the cumulative FOI up to age *a* will have acted on susceptible hosts. The probability of past infection is thus the cumulative distribution function of the time to infection (or 1 – the survival function). The underlying idea here is that any given individual of age *a* will have experienced a cumulative FOI throughout its lifetime, represented by the integral in Equation (2). The exponential term thus represents the probability of being uninfected and its complement (Equation 2), is thus the probability of being infected by age *a*. The parameters of Equation (2) can be estimated from the observed age-prevalence data recording the change in function *P*(a) with age (see Text S1 for detailed R code).

We use the so called ‘piece-wise constant’ model to estimate age-dependent λ(a), which assumes a fixed FOI within pre-determined age intervals which should be inspired by some prior knowledge of age-heterogeneities in the population of interest. For example, school-age versus pre-school children and measles dynamics [11]. We chose a 3 age-interval model which corresponds to the hypothesized routes of transmission (see Figure 1) and has a comparable expected number of events in each interval (i.e. ∼ equal numbers of rabbits in each of the 3 age-classes) which is important for model integrity [36]. It should be noted that in our initial explorations we used a flexible piecewise constant model for *λ*(a) with 8 age-intervals (in months) [0, 1), [1, 4), [4, 8), [8, 12), [12, 18), [18, 22), [22, 26) and [26+), but such a highly segmented model was ill-determined and is not reported here. The probability that a subject has converted by age *a* is *P*(*a*), and the probability that the subject has not converted is 1 - *P*(*a*). We use maximum likelihood techniques to find values of FOI elements that best fit the age-structured prevalence data. The individual infection status is considered a binary outcome variable *Y*, with *Y* = 1 for infected- and 0 for uninfected-individuals and we assume a conditional Bernoulli likelihood for the binary outcome variable (i.e. disease incidence), where the probability, *P,* of an individual being infected at age *a* is:
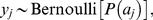
(3)where *y_j_* denotes the infectious status of the *j*th individual. We can estimate constant λ(*a*) the age-varying FOI by integrating equation (2) and numerically maximize the likelihood of the coefficients according to equation (3). This is straight forward within the R statistical programming language (http://www.R-project.org). The detailed numerical recipe is given in the R code Text S1. We use the quasi-Newton method to estimate age-varying λ(a) by minimizing the negative log-likelihood. We computed standard errors using partial profile likelihood (see Text S1 and [37]).

For the hypothesized transmission routes (*a*)–(*c*) (see Figure 1) we predict the FOI curve to peak at young, intermediate and high age-classes, respectively, whereas (*d*) should result in an age-invariant curve. We tested these predictions using the FOI estimates and their associated standard errors and used Akaike's Information Criterion (AIC) and the ΔAIC statistic for model comparison [38].

#### (ii) Generalized linear models (GLMs) and generalized linear mixed models (GLMMs)

To test for evidence of significant sibling-to-sibling transmission we used a binomial regression to investigate whether the infection status of co-housed siblings could help explain disease transmission events (see Sampling Strategy Two in M&M for data collection details and Text S1 for R code). Transition of a previously uninfected animal to infected within a sibling pair during their time of co-housing (4 weeks) was merited a ‘disease conversion’ event and scored ‘1’, with no change in infection status scored a ‘0’.

The fate of siblings in the mother-offspring cages is unlikely to be independent (see Sampling Strategy Three). For example, if mother-offspring infection is important, all offspring will have a simultaneously increased risk during periods of enhanced maternal shedding. Indeed most litter-based transmission events should result in significant correlation among the fate of siblings. Testing for such correlation is therefore important in its own right. However, additionally non-independence of siblings violates a basic assumption of generalized linear regression. We can deal with the statistical challenge of the non-independence by modeling litter-membership as a random effect in a generalized linear mixed-model (see [21] for a general introduction to GLMM's for biologists). Specifically we used random effect binomial regression analysis (with a complementary log-log link) with litter as a random variable. The algorithm we used for fitting this model was Penalized quasi-likelihood (PQL) as implemented in the *glmmPQL*-function of MASS R-package [39] (see Text S1 for more details and R code). The between-litter random effect is assumed to follow a multivariate normal distribution. Qualitative differences due to age, sex and date of sampling were consistent across rabbitry buildings A–C and quantitative differences were controlled for by including facility building as a factor in our analyses. For all analyses, maximal models were first fit to the data and minimal models then obtained by removing non-significant terms, beginning with the interaction terms (with *p* = 0.05 as a threshold), to arrive at the final models for which parameter estimates are reported.

## Results

### Force-of-infection

**Figure 2:**
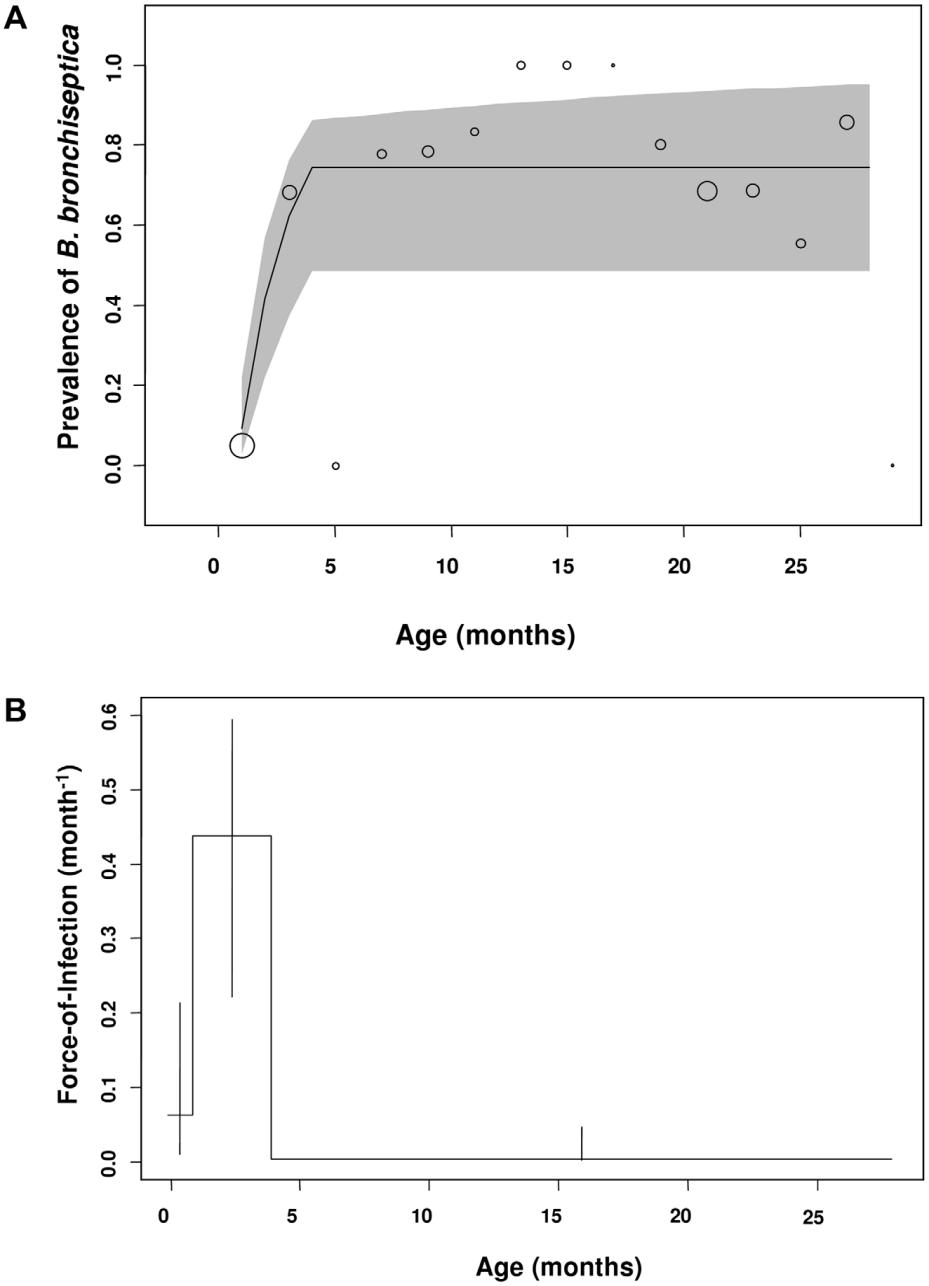
Estimated and observed prevalence of *B. bronchiseptica* and the force-of-infection estimated in the commercial rabbitry. (*a*) Observed (bubble plots) and expected (line graph) cumulative proportions infected by age (*P*(a)) in equation 1. (*b*) The fitted force-of-infection (λ(a)) in equation 1. The percentage of rabbits infected in each age-class is presented as a bubble whose size is proportional to the sample size; thus, as FOI estimates were based on data from 214 rabbits, the bubble at 1 month of age for example, represents 59 rabbits. 95% confidence intervals were computed using partial profile likelihood [37].

Two different catalytic models were fitted to the data: a piecewise constant FOI with 3 age-intervals (Figure 2b), corresponding to the hypothesized routes of transmission outlined in the Introduction and Figure 1 and a constant FOI corresponding to the null hypothesis. The 3 age-interval model fits the data better (having the lowest AIC value of 219.39) than the null hypothesis of a constant FOI (ΔAIC of model (*c*) versus (*a*)  = 39.6). The model results show close correspondence between the observed and expected prevalence data (Figure 2a). Both data and model fits exhibit a rapid increase in prevalence during the first and second age-classes (i.e. in rabbits up to 5 months; Figure 2a). During the first month of life, the estimated FOI is substantial (Figure 2b; FOI = 0.16 month^−1^) and peaks in the second age-class (Figure 2b; FOI = 0.20 month^−1^), with the older age classes (from 5–30 months of age) having the lowest FOI estimates (Figure 2b; from 3.3×10^−4^ to virtually zero).

**Figure 3:**
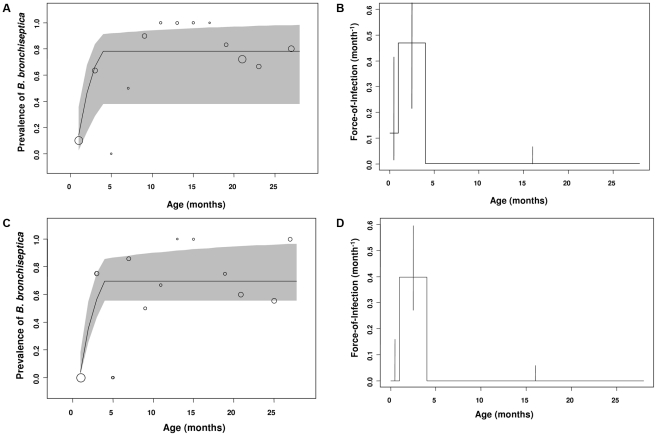
Gender differences in *B. bronchiseptica* prevalence and the force-of-infection estimated in the commercial rabbitry. Estimated and observed prevalence of *B. bronchiseptica* in (*a*) female and (*c*) male rabbits. The force-of-infection in (*b*) female and (*d*) male rabbits. The percentage of rabbits infected in each age class is presented as a bubble whose size is proportional to the sample size and standard errors were computed using partial profile likelihood [37].

Next we examined whether gender differences existed for FOI estimates. No differences between sexes were found in the FOI estimates. In the younger age-classes, the prevalence data and model estimates peaked in the second age-class resulting in positive FOI estimates in young weaned kits (1 to 4 month olds: Figure 3a–d). In the older age-classes, *B. bronchiseptica* prevalence asymptoted and subsequently fell for both sexes, with a concomitant decline in the FOI estimates (Figure 3a–d).

### Sibling versus maternal routes of infection

**Figure 4:**
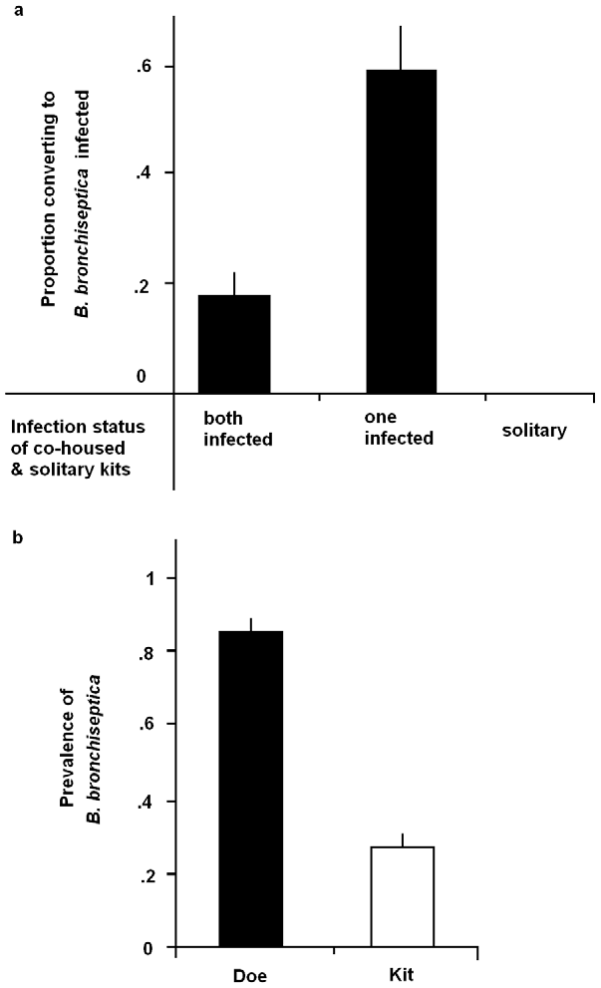
Sibling and maternal infection routes. (*a*) The proportion of uninfected sibling rabbits converting to *B. bronchiseptica* positive upon co-housing with an infected sibling and (*b*), *B. bronchiseptica* prevalence in does and kits at time of weaning. Bars represent mean ± S.E.M.

To examine the likelihood of becoming infected from an infected sibling, we ran a binomial regression on the experimental sibling-to-sibling transmission experiment (see Sampling Strategy Two in M&M for details). Being co-housed with an infected sibling increased the probability of becoming *B. bronchiseptica* positive (Figure 4a: co-housed with infected sibling: Z = 2.42, *p* = 0.016), such that uninfected kits were 3.85 times more likely to become infected when they were co-housed with an infected- compared to an uninfected-kit (Figure 4a: 95% C.I. for odds ratio 3.85: 1.29–11.46). None of the solitary *Bordetella*-free rabbits (housed alone in isolation) converted to disease-positive during this time.

Using the maternal transmission data (Sampling Strategy Three in M&M for details), the importance of sibling-to-sibling versus mother-offspring routes of transmission was investigated. First, the data revealed substantial correlation (0.53) among the infection fate of siblings and a highly significant litter-random effect (litter variance  = 4.2±0.7), demonstrating the importance of within-litter transmission. Although the prevalence of *B. bronchiseptica* was significantly higher in does compared to kits (Figure 4b; Z = 5.03 *p*<0.0001), having an infected mother did not significantly increase the probability of kits being infected (infected mother: Z = 1.74, *p* = 0.09). Nor was there any significant relationship between the litter random effect and the mothers prevalence status (Z = −1.05×10^−15^, *p* = 1.0).

### *Bordetella bronchiseptica* diversity

We used MLST analysis to characterize the relationship between 90 isolates collected from four sampling efforts across rabbitry buildings. All isolates were of sequence type (ST)-14, which is a member of the *B. bronchiseptica* complex I [35]. Thus, one circulating strain appears to dominate in our rabbit population.

## Discussion

This study demonstrates how FOI estimates coupled with random effects binomial regression analyses represent powerful tools for discerning between alternative modes of transmission for a directly transmitted pathogen. Specifically, our results support a role for sibling-to-sibling transmission among young weaned kits as a major route of *B. bronchiseptica* spread in the rabbit population studied (Figure 2). That the FOI reached a maximum value between 1 to 4 months of age – a time period when kits are re-housed in sibling pairs – followed by a sharp decline in the older age-classes, is consistent with high between-sibling transmission in young weanlings (Figure 2), regardless of host sex (Figure 3). Results from the binomial regression analyses further support a major role for sibling-to-sibling transmission in driving *B. bronchiseptica* dynamics in the rabbitry; being co-housed with an infected sibling increased the risk of infection almost 4-fold (Figure 4a). In comparison, the data did not support all other potential transmission routes; namely maternal, breeder or environmental routes. These insights shed light on the dynamics of disease spread and allow an assessment to be made of the best method(s) for effective long-term disease control, discussed more fully below.

A basic motivation for this study was to demonstrate how robust statistical tools can be used to disentangle routes and modes of transmission in humans and social animals from infection-at-age data (within family groups), which is of broad medical, ecological and veterinary interest. The FOI analyses we present may have greatest application for analyzing disease dynamics in medical and agricultural settings because here one often has direct access to date-of-birth information, knowledge of the distinct mixing patterns over the lifetime of the host, as well as host infection status (for example, by detecting a serological response in the live animal, by the polymerase chain reaction (PCR) or by pathogen isolation). One complexity which often arises in analyses of medical and agricultural diseases is clustering in the data; hosts live in families, litters or herds and once an infection is introduced, hosts within that cluster have a higher instantaneous rate of becoming infected than those outside the cluster. Our use of random effect binomial regression analysis allows us to estimate the subject-specific measure of the effect [20] and evaluate the importance of social mixing in disease spread. Thus, using the following protocol, the transmission dynamics of a range of directly transmitted infections can be analyzed by: (1) using the catalytic model and associated FOI analysis to determine the core susceptible age-class(es); (2) using random-effect binomial regression to inform on whether transmission is largely within the social group (family/litter/herd etc) or from an external social group; (3) carefully constructing transmission experiments, whenever possible, to test whether within-group versus between-group individuals are the dominant source of infection.

What might explain heterogeneities in rabbit susceptibility to *B. bronchiseptica* infection; for example, the decline in *B. bronchiseptica* prevalence in older-age classes (in rabbits ∼20 months of age)? Between-rabbit variation in protective anti-*B. bronchiseptica* immunity – and hence resistance to infection – is likely to at least partly explain differences in host susceptibility to infection. Indeed, recent work has shown that the protective immune response against *B. bronchiseptica* varies between individual rabbits, with robust serum IgG detected in some hosts for up to 5 months post infection, which correlated with clearance from the respiratory tract [28]. Given the persistent nature of *B. bronchiseptica* infections in rabbits – infections of 5 months were routinely recorded [28] – and other mammals [23], the decline in prevalence we observe is unlikely to be driven by bacteria clearance and recovery. Rather, some level of enhanced immune protection in older age-classes may be responsible for conferring some level of anti-bordetella resistance. Thus, the low attack rates (or number of reported cases per unit time in a given age-class, divided by the number in that age class) in older-age classes likely reflect low proportions of rabbits *susceptible* to infection – i.e. immune, disease-negative hosts – rather than a real decline in the rate at which susceptible rabbits acquire infection. In addition, between-rabbit heterogeneities in protective anti- *B. bronchiseptica* immunity might also help explain differences in rabbit susceptibility to infection in the maternal- and co-housed sibling- transmission studies reported here.

Is there any epidemiological support for the major route of *B. bronchiseptica* spread (sibling-to-sibling) identified using our statistical framework? *B. bronchiseptica* is known to pass efficiently and spread rapidly between populations of young weaned pigs [40], consistent with a sibling-to-sibling route for *B. bronchiseptica* transmission amongst young farmed animals. This would be particularly true in agricultural systems where an all-in/all-out (the facility is completely emptied and cleaned between groups of age-matched animals which move together between phases of production) policy of animal breeding is not practised, as is the case in the rabbitry under study. However, that our FOI estimates were above 0.1 before 1 month of age suggests some maternal or environmental transmission is occurring in young weanlings and may be key to initiating the sibling-to-sibling transmission which follows. Indeed, a maternal route of transmission is thought initiate *B. bronchiseptica* infections in swine and rabbits [26], [27], but that infection only becomes endemic when passed horizontally between different batches of susceptible young [27]. Interestingly, the time when FOI values peaked in young weanlings, coincided with a period where maternal protection wanes in kits – antibodies against *B. bronchiseptica* decreased between 2- 6 weeks of age in rabbits [25] – and could also contribute to increased susceptibility to infection observed in this age class. Thus, based on our findings and the published literature, we propose that the cycle of *B. bronchiseptica* infection in our rabbitry is maintained by a proportion of chronically infected breeder females and males (the infectious reservoir) with the majority of transmission occurring between young weaned siblings.

One important application for the analytical tools presented here is in the implementation of targeted disease control programs. Given that targeting those high-risk subgroups identified as playing key roles in transmission – rather than applying disease control measures randomly – is one efficient strategy to control disease [2], [6], a precautionary management approach might rely on the selective removal of infected weanlings to reduce sibling-to-sibling transmission. Selective removal of breeder animals – which may represent potential maintenance hosts for *B. bronchiseptica* – may also improve disease control by eliminating the infectious reservoir. Indeed, pre-emptive culling based on pre-determined patterns of disease spread has been successfully used to combat the spread of foot-and-mouth disease in cattle [41], [42]. The relationship between culling intensity and the resulting disease prevalence can be estimated when knowledge on population density and disease prevalence is available [43]. This allows estimates to be made regarding the level of culling needed to produce significant reductions in disease prevalence.

The analyses presented here can be applied to a range of medical and veterinary diseases to better understand the dynamics and mechanisms of disease spread, provided they are directly transmitted and induce lifelong immunity to re-infection. For example, the disease caused by mycobacterium – the etiological agent of tuberculosis in animals including bovine and humans – is largely directly transmitted, causes a sub-acute or chronic disease state which is irreversible [14], [15], [18] and can be routinely confirmed via culture, making it a tractable disease for application of FOI analyses. Indeed, the tools of infectious disease quantitative epidemiology have successfully been applied to further understand *Mycobacterium bovis* infection dynamics in wildlife population of badgers [44], ferrets [14] and bison [15], [45] and has shed light on likely patterns of mycobacteria transmission in the wild. However, these tools have not been used to the same effect in agricultural settings despite the debilitating effects of this disease and the potential to improve disease control therein. Other veterinary diseases which are tractable for this type of analyses include brucellosis, bovine herpes infection, classical swine fever, bovine mastitis and atrophic rhinitis in swine, to name but a few. Finally, the FOI model presented here can be extended to include diseases with reversion to non-diseased state or non-benign diseases (i.e. associated with increasing death rate), or indeed to include a period where hosts are not exposed to infection (for example, when maternal antibodies are known to provide protection against specific diseases early in life) similar to a guarantee time in survival analysis (see Caley & Hone 2002 for examples of such extensions).

Our study has some limitations. Although the method we outline can clearly reveal the age-class for which most of the new infection occurs, it cannot easily discern whether that infection is mainly within an age-class versus from a different age-class. However, once the high FOI age-class is identified, careful design of transmission experiments could confirm the likely source of infection, and such studies are underway in our University. To control and possibly eradicate infectious diseases we need a better understanding of pathogen population dynamics and structure. Indeed, only when HIV population structure was understood did the requirement for a three-cocktail HIV drug therapy become clear [46]. Knowledge of pathogen population structure is also needed to determine which disease-associated genes are under directional selection change. To this end we used MLST analysis to investigate whether strain-specific differences existed in the epidemiological pattern of infection [30], [31]. However, only one major circulating sequence type – ST14 – was identified in our rabbits regardless of rabbit age, sex or facility building. The dominance of ST14 across our facility may be due to the successful expansion of this single serotype over time. Alternatively, a limitation in sampling could have potentially biased our results; the sequence type of only 1 colony per swabbed plate (i.e. per rabbit) was determined at each sampling round. Therefore if the rabbit was colonized with multiple strains we most likely detected the dominant type (ST14). More intensive sequencing typing is required to test whether the lack of genetic variation we report is real and such studies are ongoing.

This study demonstrates the ease with which potential routes and reservoirs of infection can be discriminated amongst from age-prevalence data in medical, agricultural, and wildlife setting when we have access to fundamental age-prevalence data. Much remains to be done to achieve a better understanding of the complex dynamics of chronic infections and to extend this model to incorporate factors such as host immunity and parasite genetic variation.

## Supporting Information

Text S1 R code_Long et al. Completely annotated R code for FOI and GLM statistical analyses.
(0.06 MB PDF)
