# Supplementary material for: Identifying the Age Cohort Responsible for Transmission in a Natural Outbreak of Bordetella bronchiseptica
Source: PLoS Pathog. 2010 Dec 16;6(12):e1001224. doi: 10.1371/journal.ppat.1001224 (PMC3002977; doi:10.1371/journal.ppat.1001224)
Supplement: Text S1 — R code_Long et al. Completely annotated R code for FOI and GLM statistical analyses. (0.06 MB PDF) [file ppat.1001224.s001.pdf]

S1. R code_Long et al

Estimating the force-of-infection

Here, observed age-specific prevalence data (n = 214 rabbits) was collected from nasal swabs using the data set resulting from "Sampling strategy one: force-of-infection" (see Materials and Methods for details). This data represents interval-censored infection-time data, such that each individual is either infected (Y=1) or not (Y=0) within a set interval of time. For a non-immunizing persistent infection such as *B. bronchiseptica,* (see key assumptions of force-of-infection (FOI) models in the Statistical Analysis, M&M) the age-specific prevalence of, *P(a),* can be estimated via the catalytic model:

$$P(a) = 1 - \exp\left(-\int_0^a \lambda(a)da\right) \tag{S1}$$

where λ(a) is what mathematical epidemiologists call the age-dependent FOI and what statisticians would call the age-dependent hazard (Hens, Aerts et al. ; Muench 1959). Assuming that the infection hazard is invariant with respect to age is often unrealistic (see Statistical Analysis, M&M). To incorporate age-dependency we use the piece-wise constant parametric model where, for pre-determined intervals, a constant FOI is assumed. Interval choice is based on some prior knowledge of age-classes of mixing cohorts in the population. When the FOI is assumed to be piece-wise constant across k age classes and each segment has a starting age l_a and duration d_k, the integrand in equation (S1) for an individual whose age lies within the k'th age class will be:

$$\int_0^a \lambda(a)da = \lambda_k(a - l_k) + \sum_{a=0}^{k-1} \lambda_a d_a \ . \tag{S2}$$

We estimate the age-class-specific hazards by maximum likelihood. Algorithmically, we can note that the Bernoulli distribution (Equation 3 in main text) is identical to a binomial distribution with a sample size of 1. The function `dbinom()` in R evaluates the binomial

likelihood. We will need to do numerical minimization of the negative log-likelihood for our analysis. To do this we first write a general function that takes candidate values for the piecewise-constant FOI as an input (par), the dataset to be analyzed (data) and the lower cut-off for each age-class (cate), which provides the value of the negative log-likelihood as output. The function code is:

```
loglikpc=function(par,data, cate){
   dur=c(diff(cate), 0)
   ll=0
        for(a in 1:length(data$age)){
         dummy1=data$age[a]>cate
         dummy2 = data$age[a]>cate & !c(data$age[a]>cate[-1], FALSE)
         dummy1=c(data$age[a]>cate, FALSE)[-1]
         inte=sum(dur*exp(par)*dummy1)+
                 exp(par[dummy2])*(data$age[a]-cate[dummy2])
         p=1-exp(-inte)
        ll=ll+dbinom(data$sick[a],1,p,log=T)
         }
 return(-ll)
 }
```

The inner working of this function is as follows: line 1 calculates the duration of each age class (dur); line 2 sets the initial log-likelihood to zero; lines 3 – 9 is a loop that for each individual in the data-set, calculates the integrand corresponding to equation S2 (inte), evaluates the prediction from the catalytic model (p) and finally adds the log-likelihood for each individual; finally, line 11 outputs the negative log-likelihood. Note, that to ensure that the FOI values are positive we actually estimate the log-values.

The data-set needs to be formatted so that each individual is a unique line, the column labeled ‘age’ gives the age of the individual, the column labeled ‘sick’ has a 0 (zero) for

healthy individuals and 1 (one) for infected individuals. In the example below the dataset is named `dat`.

With this setup, we can now estimate λ(a) by minimizing the negative log-likelihood using the quasi-Newton 'BFGS' method (for Broyden, 1969; Fletcher, 1970; Goldfarb, 1970 and Shanno, 1970) - generally regarded as the best performing method - as implemented in the `optim()`-function of R.

First we define the vector giving the cut-off ages. We call this vector x:

x=c(0, 1, 4)

Then we provide some (arbitrary) initial values for the log-transformed age-specific FOI values. We call this vector para:

para=log(c(0.1, 0.1, 0.1))

Finally, we call the numerical optimizer in R and save the output as `est`:

est = optim(par=log(para),fn=loglikpc,cate=x, method="BFGS",   data=dat, control=list(trace=2, maxit=1000))

The maximum likelihood estimates for the log FOI is given in `est$par`.

Statistical uncertainty*

Because of strong multi-colinearity among the λ_a-estimates, we use partial profile likelihoods to erect confidence intervals (Diggle 2006). That is, we profile the likelihood for each segment separately, maximizing the likelihood with respect to the other segments. This step is computationally expensive and a bit technical.

We first modify our likelihood function to flag the segment to be profiled (which) and what value to consider for that segment (wval). The modified function is:

```
loglikpc2=function(par,data, cate=x, which, wval){
dur=c(diff(cate), 0)
ll=0
for(a in 1:length(data$age)){
        dummy1=data$age[a]>cate
        dummy2 = data$age[a]>cate & !c(data$age[a]>cate[-1], FALSE)
        dummy1=c(data$age[a]>cate, FALSE)[-1]
        par[which]=wval
        inte=sum(dur*exp(par)*dummy1)+exp(par[dummy2])*(data$age[a]-
        cate[dummy2])
        p=1-exp(-inte)
        ll=ll+dbinom(data$sick[a],1,p,log=T)
        }
return(-ll)
}
```

Again we define the vector giving the cut-off ages.

```
x=c(0, 1, 4)
```

Then provide some (arbitrary) initial values for the log-transformed age-specific FOI values:

```
para=log(c(0.1, 0.1, 0.1))
```

Next we set up a grid (in this case a sequence of length 31) of candidate values (on a log-scale) to consider (wval):

wval=seq(-15, 0, by=.5),

and a empty vector to store the partial profile negative log likelihood values (this example profiles segment 1 so the segment is named ll1):

ll1=rep(NA, 31)

Then we call the numerical optimizer in R repeatedly (31 times in this example) and save partial profile likelihood values in ll1:

```
for(i in 1:31){
        tmp=optim(par=log(para),fn=loglikpc2,cate=x, method="BFGS",
        hessian=TRUE, data=dat,control=list(maxit=1000), which=1, wval=wval[i])
        ll1[i]=tmp$value
}
```

Finally, we use a smoothing spline to interpolate the partial likelihood profile:

```
tmp2=smooth.spline(wval,ll1)
new=seq(-15,0, by=.001)
interp= predict(tmp2, new)$y
```

and then use the fact that the profile likelihood is χ^2-distributed to erect 95% confidence intervals:

```
mle1=new[which.min(interp)]
tmp3=(predict(tmp2, new)$y-min(predict(tmp2, new)$y))-qchisq(0.95,1)
range(new[tmp3<0])
```

The final *range()* call provides the 95% confidence interval.

Generalized linear models (GLMs)

To test for evidence of significant sibling-to-sibling transmission we used binomial regression with a complementary log-log link. We use the complementary log-log link, here, because the resultant parameter estimates then has a hazard (i.e. FOI) interpretation, as opposed to the 'odds' interpretation that would result from the commonly used logistic ('logit') link . This was done using the data set resulting from "Sampling strategy two: sibling-to-sibling transmission" (see Sampling Strategies in M&M for details). This data set (n = 160 kits total) comprised of a column entitled: '*disease_conversion*', which represents the binary variable that denotes whether or not a co-housed susceptible sibling was infected at the end of the experiment; '*co-housed status*', which informed on infection status of co-housed siblings on initiation of experiment; 'facility', to control for possible differences between the two breeding houses and 'family', to control for possible cohort effects. After importing the data into R (Crawley 2007), the GLM was run as follows:

glm(disease_conversion ~ cohoused_status + facility, family = binomial(link = "cloglog"))

Generalized linear mixed models (GLMMs)

Next, to investigate if (a) offspring of infected mothers have an increased instantaneous risk of becoming infected and (b) if offspring of the same litter tended to have the same infection fate because of within-litter transmission, we used random effect ("generalized linear mixed model (GLMM)") binomial regression, with litter as a random variable using the data set resulting from "Sampling strategy three: maternal transmission" (see Materials and Methods, 'Sampling Strategies' section for details). This data set (with 86 does and 408 kits) has a column entitled '*disease_conversion*' representing the binary variable that flags whether or not any given susceptible offspring was infected at the end of the experiment, '*mother_infected*' representing the binary variable that flags whether or not the mother was infected before kit birth. Breeding facility and litter size were also included as covariates to control for any possible effect of breeding house or litter size, respectively. The algorithm we used for fitting this model was Penalized quasi-likelihood

(PQL) as implemented in the *glmmPQL*-function of MASS R-package. First, we attach the necessary package into R:

```
require(MASS)
```

We then fit the model. Again we use the complementary log-log link, here, because the resultant parameter estimates retains a hazard (i.e. FOI) interpretation,

```
fit2 = glmmPQL(disease_conversion ~ mother_infected + litter_size + facility,
      random = ~1 | Litter, family = binomial(link = "cloglog"))
```

The litter random effect is specified by the *random = ~1 | Litter* argument. See (Bolker, Brooks et al. 2009) for a general introduction to GLMM's for biologists. Finally we can formally test for significantly enhanced FOI of offspring of infected mothers by investigating the estimated litter-level random effect:

```
> x=random.effects(fit2)[,1][mother_infected ==0]
> y=random.effects(fit2)[,1][ mother_infected ==1]

> t.test(x,y)
```

References

Bolker, B. M., M. E. Brooks, et al. (2009). "Generalized Linear Mixed Models: a Practical Guide for Ecology and Evolution." Trends Ecol Evol 24(3): 127-135.

Broyden, C.G., 1969. "A new double-rank minimization algorithm", *Notices of the American Mathematical Society*, 16:670.

Crawley, M. J. (2007). The R Book: West Sussex, UK: John Wiley & Sons Ltd.

Diggle, P. J. (2006). "Spatio-temporal point processes, partial likelihood, foot and mouth disease." Stat Methods Med Res 15(4): 325-36.

Fletcher, R., 1970. "A new approach to variable metric methods", *Computer Journal*, 13:317-322.

Goldfarb, D., 1970. "A family of variable metric methods derived by variational means", *Mathematics of Computation*, 24:23-26.

Hens, N., M. Aerts, et al. "Seventy-five years of estimating the force of infection from current status data." Epidemiol Infect 138(6): 802-12.

Muench, H. (1959). "Catalytic Models in Epidemiology." Harvard University Press.

Shanno, D.F., 1970. "Conditioning of quasi-Newton methods for function minimization", *Mathematics of Computation*, 24:145-160.
